# Editorial: Lifestyle interventions for traumatic stress (LIFTS)

**DOI:** 10.3389/fpsyg.2024.1367344

**Published:** 2024-01-23

**Authors:** James W. Whitworth, Erica R. Checko, Simon Rosenbaum

**Affiliations:** ^1^National Center for PTSD, VA Boston Healthcare System, Boston, MA, United States; ^2^Boston University Chobanian & Avedisian School of Medicine, Boston, MA, United States; ^3^Discipline of Psychiatry and Mental Health, Faculty of Medicine and Health, University of New South Wales, Sydney, NSW, Australia

**Keywords:** PTSD, exercise, mental health, lifestyle intervention, physical activity, randomized controlled trial (RCT), depression, trauma

## Introduction

Globally, treatment of poor mental health including psychological trauma is at a precipice. Long waiting lists associated with a lack of trained providers and high costs of treatment mean most people affected rarely receive adequate, evidence-based treatments. Among those who do, treatment options rarely fully alleviate symptoms, while a concurrent decline in physical health among people exposed to trauma, is too often accepted as inevitable—referred to as a form of “therapeutic nihilism.” Our current approaches to treating the psychological consequences of trauma exposure fail to routinely address the systemic impacts within the body, despite evidence of demand for and acceptability of body and lifestyle-focused interventions. One can simply look at the remarkable success of Besel Van Der Kolks “*the body keeps the score*,” which has sold roughly ~2 million copies and spent more than 150 weeks on the New York Times best seller list for paperback non-fiction (Haslam, 2024).

Psychological trauma and posttraumatic stress disorder (PTSD) have profound negative effects on biopsychosocial functioning (American Psychiatric Association, 2013) and increase the risk for chronic disease and disability (Kibler et al., 2014; Wolf et al., 2016). Current evidence-based treatments are effective, yet often do not fully alleviate trauma symptoms, are unappealing to many trauma survivors, or are unavailable due to barriers, such as cost or access to trained providers (Kantor et al., 2017). Moreover, current frontline treatments rarely address trauma-related maladaptive changes in lifestyle (e.g., physical inactivity and poor diet) that influence physical and mental health, and risk of mortality. Yet, interest in lifestyle-based interventions for PTSD (Pebole et al., 2022, 2023) and related issues (e.g., depression and substance use) (Abrantes et al., 2011; Busch et al., 2016) is high among those living with these conditions.

The lack of focus on lifestyle behaviors in frontline treatments for trauma survivors is not surprising, given little attention given to them in the current treatment guidelines. Specifically, the American Psychological Association (APA) makes no mention of physical activity, exercise, or sport within their most recent treatment guidelines for PTSD (American Psychological Association, 2017). It is unclear if these interventions were even considered for evaluation. The International Society of Traumatic Stress Studies (ISTSS) does better and acknowledges the field, but states there is “Insufficient evidence to recommend” exercise as a part of PTSD treatment (International Society of Traumatic Stress Studies, 2018), despite meta-analytic data supporting the opposite (Björkman and Ekblom, 2021; Ramos-Sanchez et al., 2021). Resistance to the role of physical activity-based lifestyle interventions for mental health is not new. For years the value of physical activity for depression has been downplayed (Ekkekakis and Murri, 2017), despite the abundance research demonstrating its effects are comparable to many first-line treatments (Cooney et al., 2013; Ekkekakis, 2015).

It is for these reasons, the present Research Topic *Lifestyle interventions for traumatic stress* (*LIFTS*) sought to provide a platform to promote and disseminate high quality lifestyle interventions and related research for PTSD and/or common medical and psychiatric comorbidities of PTSD (e.g., depression, anxiety, and insomnia) for which lifestyle interventions/health behavior change can help to manage or improve. We hope to facilitate greater awareness for these treatment modalities among clinicians, patients, policymakers, and other key stakeholders.

## Preview of the included research

### Sport and recreation

Walter, Otis, Hose et al. provide excellent rationale for the value for sport and recreation research for trauma survivors, “Recreational and adaptive sports programs may be beneficial therapeutic interventions for improving psychological outcomes among veterans and service members with PTSD because they provide opportunities for exercise, socialization, respite, and time outdoors.” Results from an annual, week-long adaptive sports program suggest participation in such a program can lead to significant reductions in PTSD symptom severity and anxiety. Further, results from a randomized controlled trial demonstrates both surfing and hiking activities improve depression symptoms among veterans with high rates of PTSD (Walter, Otis, Miggantz et al.).

### Combining physical activity with trauma-focused treatments

Recent research suggests augmentation of trauma-focused therapy with aerobic exercise may enhance the therapeutic effect of these treatments (Bryant et al., 2023; Crombie et al., 2023). However, these augmented benefits may not apply to all physical activity interventions. In a randomized controlled trial comparing a physical activity enhanced trauma-focused treatment program to trauma-focused treatment alone, Voorendonk et al. found no evidence of physical activity-based enhancements. In another randomized controlled study combining aerobic exercise with trauma-focused treatments for PTSD among active-duty service members, Young-McCaughan et al. found that exercise reduced insomnia symptoms, relative to non-exercise controls but found no additional benefit to PTSD symptoms. These important studies highlight the need for additional research into specific mechanisms (e.g., timing of physical activity/exercise interventions and dose parameters, such as intensity, duration, frequency, and mode of activity) and commonly co-occurring conditions (e.g., insomnia) that maximize the benefits of physical activity for trauma survivors.

### A qualitative lens

To date, most lifestyle and trauma research uses quantitative methods. Within are two qualitative studies that provide much needed context to the field. The first study is a mixed-methods study of trauma survivors that seeks to understand what is needed to develop a survivor-informed evidence-based weightlifting program (Vigue et al.). The second study interviewed trauma researchers, clinicians, and professionals to better understand current practices, gaps and/or blind spots within the field (Hira et al.). Both studies enrich current quantitative efforts within the field and provide valuable insights from both providers and survivors that can be used in treatment development and improving patient experiences/outcomes.

### Addressing the physical and mental health needs of people experiencing homelessness

Lifestyle intervention and trauma research is not just about reducing symptom severity. Vickery et al. discuss pilot feasibility data and propose a novel lifestyle intervention, “…designed for people experiencing homelessness with type 2 diabetes and address health equity gaps in people who have experienced trauma.” Their intervention includes elements of behavioral activation, lay health coaching programs for diabetes and resources from Health Care for the Homeless. Importantly, its development was informed and vetted by affected persons and providers.

## Concluding thoughts

The following Research Topic contains an impressive array LIFTS research from an international body of experts. This Research Topic of studies highlights the value and importance of lifestyle interventions in trauma treatment paradigms. However, this research and the lukewarm reception of lifestyle interventions by APA, ISTSS and other governing bodies also underscore the need for further high-quality research to identify critical mechanisms of action, develop evidence-based best practices, and effective implementation/dissemination strategies. Thus, this should serve as a call to action for all LIFTS researchers, clinicians, and community members. Please join us as other LIFTS community members as we move the field forward by following us @LIFTS_SIG on Twitter/X.

## Author contributions

JW: Writing – original draft, Writing – review & editing. EC: Writing – original draft, Writing – review & editing. SR: Writing – original draft, Writing – review & editing.
